# Technological Advances in Healthcare and Medical Deontology: Towards a Hybrid Clinical Methodology

**DOI:** 10.3390/healthcare13141665

**Published:** 2025-07-10

**Authors:** Vittoradolfo Tambone, Laura Leondina Campanozzi, Lucio Di Mauro, Fabio Fenato, Guido Travaini, Francesco De Micco, Alberto Blandino, Giuseppe Vetrugno, Giulia Mercuri, Mario Picozzi, Raffaella Rinaldi, Francesco Introna

**Affiliations:** 1 Research Unit of Bioethics and Humanities, Department of Medicine and Surgery, Università Campus Bio-Medico di Roma, 00128 Rome, Italy; v.tambone@unicampus.it (V.T.); f.demicco@policlinicocampus.it (F.D.M.); 2 Legal Medicine, Department of Medical, Surgical and Advanced Technologies “G.F. Ingrassia”, University of Catania, 95123 Catania, Italy; dr.luciodimauro@gmail.com; 3 Società Italiana di Medicina Legale e delle Assicurazioni (SIMLA), 00161 Rome, Italy; ffenat65@gmail.com; 4 Faculty of Medicine, University of Vita-Salute San Raffaele, 20132 Milan, Italy; travaini.guido@hsr.it (G.T.); blandino.alberto@hsr.it (A.B.); 5 Department of Clinical Affair, Fondazione Policlinico Universitario Campus Bio-Medico, 00128 Rome, Italy; 6 Department of Health Care Surveillance and Bioethics, Section of Legal Medicine, Università Cattolica del Sacro Cuore, 00168 Rome, Italy; giuseppe.vetrugno@policlinicogemelli.it (G.V.); giulia.mercuri@unicatt.it (G.M.); 7 UOS Risk Management, Fondazione Policlinico Universitario “A. Gemelli” IRCCS, 00168 Rome, Italy; 8 Research Center for Clinical Ethics, Department of Biotechnology and Life Science, Insubria University, 2100 Varese, Italy; mario.picozzi@uninsubria.it; 9 Department of Anatomical, Histological, Forensic and Orthopaedic Sciences, Sapienza University, 00189 Rome, Italy; raffa.rinaldi@uniroma1.it; 10 Department of Interdisciplinary Medicine, Section of Legal Medicine, Aldo Moro University of Bari, 70124 Bari, Italy; francesco.introna@uniba.it

**Keywords:** healthcare technology, Italian code of medical deontology, forensic medicine, professional responsibility, aware and cooperative reduction, critical medicine, informed consent, hybrid clinical methodology

## Abstract

The rapid advancements in healthcare technologies are reshaping the medical landscape, prompting a reconsideration of clinical methodologies and their ethical foundations. This article explores the need for an updated approach to medical deontology, emphasizing the transition from traditional practices to a hybrid clinical methodology that integrates both human expertise and technological innovations. With the increasing use of Artificial Intelligence, data analytics, and advanced medical tools, healthcare professionals are presented with new ethical and professional challenges. These challenges demand a reevaluation of professional responsibility, highlighting the importance of scientific evidence in decision-making while mitigating the influence of economic and ideological factors. By framing medical practice within a systemic and integrated perspective, this article proposes a model that moves beyond the reductionist and anti-reductionist dualism, fostering a more realistic understanding of healthcare. This new paradigm necessitates the evolution of the Medical Code of Ethics, integrating the concept of “medical intelligence” to address the complexities of data management and its ethical implications. The article ultimately advocates for a dynamic and adaptive approach that aligns medical practice with emerging technologies, ensuring that patient care remains person-centered and ethically grounded in a rapidly changing healthcare environment.

## 1. Introduction

The ongoing reflection on the revision of the Italian Code of Medical Deontology requires an approach that goes beyond a mere normative update, taking into account the implications related to the physician’s professional responsibility, the duty to ensure care that meets the gold standard, and the role of guarantee that the medical profession is called to uphold. In particular, it is useful to consider a perspective that also integrates the field of forensic medicine, which provides tools for a more comprehensive understanding of the challenges that physicians face. This reflective process is developing on multiple levels, driven by an increasing sensitivity towards ethical and professional responsibility, as well as the need for continuous updates to maintain a balance between technological and scientific innovation, legal obligations, and professional values.

The advent of new enabling technologies, which characterize the current industrial revolution [1], represents only a partial novelty. Tools such as Artificial Intelligence (AI), the Internet of Things (IoT), Augmented Reality, and Big Data management undoubtedly mark a significant and, in many ways, surprising technological advancement. Nevertheless, they constitute an element of a more extensive evolutionary process that possesses profound historical origins. An interesting way to grasp the impact of these innovations is through the so-called ‘Solow residual’ [2]—an economic indicator that measures the portion of productivity growth that is not explained by increases in physical capital (machinery, infrastructure) or labor but rather is attributed to technological progress. The employment of elementary linear regression models demonstrates that a considerable proportion of contemporary advancements are attributable to innovations in processes, techniques and knowledge. Although the notion of ‘technological singularity’ [3]—the point at which technology becomes capable of self-improvement without human intervention—remains more characteristic of science fiction than of contemporary scientific reality, it is indisputable that the efficacy and efficiency of contemporary technologies is already manifest. In fields such as medicine, economics, communication, and defense, the ongoing transformation is both visible and profound.

This reflection revolves around the reaction that society might have in response to the increasing spread of technologies related to Artificial Intelligence. One possible reaction could be an urgent desire to halt this wave; however, such resistance would not slow down the change; rather, it would ultimately be overwhelming and devastating. A conservative approach, evident in various contexts, emphasizes the need to build “guardrails” to limit the development of Artificial Intelligence. At the international level, several proposals for a moratorium have been put forward to slow down the pace of technological advancement. Drawing on the thinking of Imre Lakatos, who argues that scientific progress is not achieved by rejecting problematic theories, but by strategically adapting them to solve emerging problems [4], it seems that projects aimed at slowing down innovation through bans and restrictions are doomed to fail in the face of more proactive initiatives that instead seek to positively shape and guide the evolving technological reality. Another possible response would be to ‘dive’ into change without resistance, hoping to withstand its intensity ‘on apnea’, hoping that the wave will not be too strong to drown us. The most promising attitude, however, is to face the challenge with creativity and proactivity, that is, to “invent the surf”, adapting to the new realities, using the power of Artificial Intelligence as a tool to navigate rather than be overwhelmed by it. The invitation is therefore not only to try to “surf” but also to develop the skills to ride the wave, turning the challenge into an opportunity [5].

Since the advent of Homo Sapiens-Sapiens, technology has become an intrinsic product of our nature [6]. It is not merely a tool to solve problems, but a means that expresses and actualizes our humanity in profound and meaningful ways. Technology does not suppress our human essence, but rather expresses it in new forms, adapting to the challenges and opportunities of our time. In this context, a new Code of Medical Deontology should convey an attitude of openness to technological innovation, embracing technology as a valuable asset in service of best medical practices. However, this approach must go hand in hand with an increasing awareness of the “human role” of the physician, who is not merely an operator but a conscious and responsible actor in designing, developing, and using new technologies. In other words, the Code should promote a dual valorization: on one hand, the drive toward more advanced and effective technologies serving medicine, and on the other, the affirmation of a more aware and humanistic medical profession, which remains a key player in a process that is not only technical but also ethical and human. This orientation informs the exploration of two key dimensions: the normative reconfiguration of medical deontology in light of AI, and the methodological adaptation of clinical decision-making to a technologically mediated environment.

This article stems from an interdisciplinary collaboration aimed at offering a prospective and integrated understanding of the challenges posed by the integration of Artificial Intelligence in healthcare. The approach adopted integrates philosophical reflection, normative reasoning, and policy considerations, underpinned by a shared anthropological perspective of the human condition and the objectives of medicine. Instead of adhering to a rigid disciplinary framework, the paper employs a dynamic interplay between top-down and bottom-up perspectives, integrating foundational principles and regulatory frameworks with the concrete demands of clinical practice. This dual orientation reflects our conviction that regulatory innovation must be responsive to real-world experience while remaining anchored in coherent ethical and anthropological foundations.

Within this framework, this paper reflects on how the integration of AI challenges the current structure of medical deontology, calls for a revision of clinical methodology through a more dynamic and hybrid approach, and invites a rethinking of core ethical concepts—such as responsibility and autonomy—in light of the increasingly relational and technologically mediated nature of medical care.

## 2. Politics and Medicine

In the context of technological transformation, this development should mark a new chapter in medical deontology, transitioning from the implicit to the explicit [7]. This endeavor is also essential for enhancing public confidence in both medical science and politics. This section explores how the political and institutional dimension of healthcare, particularly in the context of technological transformation, necessitates a rethinking of professional responsibility, clinical standards, and the governance of knowledge. In this regard, the following points should be noted for the purposes of future reference: firstly, there is the imperative to reinterpret professional responsibility, establishing a new set of regulations based on the technological resources now at our disposal. Secondly, decisions must be based solely on scientific evidence, with economic or ideological factors having no part to play. Thirdly, the entire field of medical intelligence must be regulated transparently in order to take full advantage of new technologies. Borrowing and adapting a term from military doctrine, by the term medical intelligence we refer to the systematic collection, analysis, and interpretation of medical, bioscientific, and environmental data relevant to clinical practice and healthcare systems. In this context, the aim is to support strategic planning, ethical decision-making, and anticipatory responses in complex care environments [8].

In the field of healthcare, professionals are expected to provide the most optimal care possible to their patients, adhering to the gold standard of care. This term refers to the most effective diagnostic or treatment option available for a particular condition or disease when it is required. This approach is characterized by its reliability and accuracy and is widely accepted and recognized within the medical community. In instances where this standard is not met, the possibility of professional negligence arises [9]. It is important to note that the analysis of professional conduct is usually performed in a context unrelated to the specific circumstances present in an emergency room, operating room, or a highly complex department. The crux of the issue at hand is not to pass judgment on medical professionals who, on occasion, find themselves in situations that are challenging from an ethical, administrative, or legal standpoint. However, it is important to acknowledge the significant impact of technological advancement in the medical field on the enhancement of best practices. Consequently, it is essential to evaluate the medical responsibility of the professional environment in which doctors operate, taking into account the resources available to them.

To illustrate this point, for instance, a scenario could be considered in which four staplers are available in an operating room when six are needed: the surgeon perhaps has reported this need several times, the facility does not adequately supply the room, and the physician still has to operate. Another example could be a situation where a physician needs to do a Computed Axial Tomography (CAT) scan to assess the lung status of a COVID-19 patient but does not have the software that uses AI to optimize the reporting time, and the diagnosis comes too slowly to make the correct treatment decisions. Whilst there is still a need to refer to the physician’s professional responsibility, it is important to acknowledge the increasing role of technology in the clinical setting. This necessitates a more precise evaluation of the company’s professional responsibility, which in turn can assist in establishing a more balanced perspective on the physician’s subjective responsibility. This issue brings into focus what is commonly referred to as ‘systemic risk governance’, highlighting the critical distinction between healthcare norms and laws grounded in scientific evidence and those driven by economic or ideological considerations. When normative frameworks in health systems lack a solid foundation in science, the result is often increased systemic fragility, growing inequities, and diminished resilience.

This is a critical consideration, increasingly recognized as warranting explicit inclusion in the Italian Code of Medical Deontology. The assertion that medical practice—and its ethical codification—must be grounded solely and consistently in scientific evidence, free from economic or ideological influence, serves as both a reaffirmation and a refined articulation of principles already embedded in the Code, as reflected in Articles 3, 5, 6, 13, 15, and 16. Within this framework, Title VII of the Code assumes a clearer and more central significance. In this particular context, the act of “causing death” cannot be regarded as a scientific act, since the minimal conditions required to generate scientific evidence are absent. It is not possible to conduct clinical trials, assess the outcomes of the act, carry out follow-up evaluations, or, consequently, provide the comprehensive and adequate information necessary to obtain truly informed consent.

The management of healthcare Big Data involves the handling of highly sensitive information, which can be utilized in a variety of ways, including strategic applications such as medical intelligence. The increasing focus on Articles 10, 11, and 12 of the Code, which address professional secrecy, confidentiality, and data protection, underscores the imperative for the Code to evolve by explicitly incorporating the concept of medical intelligence through the introduction of a dedicated article. The failure to address this issue would constitute an internal contradiction. On the one hand, the importance of privacy is rightly emphasized, and strictly protected pathways are established. On the other hand, there is an acknowledgement that such data, due to their nature and potential, can be used for non-clinical purposes that raise significant ethical concerns. The inclusion of this topic in the Code would represent a significant act of transparency and modernization, as well as a crucial step in safeguarding the dignity and credibility of the medical profession in an era in which data management is becoming increasingly centralized.

These reflections on the systemic and political dimensions of medicine provide a foundation for a more focused analysis of how AI concretely affects clinical methodology and the ethical structure of care.

## 3. A Strategic Plan for Understanding the True Impact of Artificial Intelligence in the Life Sciences

This section argues that a comprehensive understanding of AI’s impact on medicine must move beyond abstract theorization to evidence-based, risk-aware experimentation. This shift is imperative to facilitate the development of a novel clinical methodology. With the approval of the Artificial Intelligence (AI) Act, institutions must ensure the safe use of Artificial Intelligence systems by citizens, prohibiting systems that present an unacceptable risk to the safety, health, dignity, or autonomy of people [10]. Hence, identifying ‘trustworthy’ AI systems is becoming a priority. Some authors have questioned the evaluation method promoted by the European Commission in the AI Act, whereby AI developers are responsible for either self-assessing their systems or submitting them to external assessments by ‘notified bodies’. People may interpret a developer’s willingness to submit an AI system to external review as a sign of trustworthiness. Therefore, citizens’ trust in AI may not derive from judgements of risk acceptability per se but be influenced by the institutional context. For this reason, it is essential to ensure the impartiality and independence of intermediaries, such as notified bodies, in order to consolidate citizens’ trust in AI [11]. Therefore, specifically for healthcare, there is an increasing ethical and professional imperative to move from theoretical reflection on AI to a systematic and objective assessment of its real impact on clinical methodology. In recent years, a large body of research has explored the relationship between AI and human health, primarily addressing overarching concerns such as core ethical principles (beneficence, non-maleficence, justice, and autonomy), explicability, legal and regulatory frameworks, algorithmic reliability, bias, equity and inclusivity, privacy and security, and the development of guidelines and best practices [12,13,14,15,16]. A layered model (technical foundations, ethical issues, and social and legal regulation) for AI governance has been previously proposed [17]. From a risk management perspective, the call for robust empirical data to evaluate the impact of AI in the life sciences highlights the crucial need for structured oversight and preventive strategies [18]. As Artificial Intelligence technologies become increasingly integrated into clinical practice, it is essential to rigorously assess potential risks—ranging from diagnostic errors to unintended consequences on patients’ mental health and the overall quality of care. This transition from conceptual analysis to structured experimentation signifies a pivotal progression in the realm of medical deontology, necessitating proactive risk identification, interdisciplinary collaboration, and systematic governance. It also introduces ethical and legal implications that demand careful governance, so that AI supports—rather than compromises—the fundamental principles of medical deontology and patient safety. In this context, risk management must evolve beyond existing models of AI application, incorporating targeted pre-training and fine-tuning that can only be achieved through a dedicated observatory and a continuously developing cloud infrastructure. This evolution is particularly relevant in fields such as disability, where AI-based tools are beginning to transform both opportunities and vulnerabilities [19]. For people with disabilities, AI offers the promise of greater autonomy and personalized interventions—but it also poses significant risks, for instance, when systems are not designed in a genuinely inclusive way or when sensitive existential data is not adequately considered. Similarly, in occupational health and safety, AI can enhance risk prediction and accident prevention, but it also demands strong safeguards to prevent new forms of surveillance, discrimination, or excessive reliance on automated decision-making. Effective risk management in these areas requires not only technical reliability but also a strong ethical commitment to equity, transparency, and human dignity.

While these areas are undoubtedly important, they often reflect a broader discourse focused on AI as it relates to the life sciences in general. To assess the specific impact of AI on clinical methodology—in care delivery, biomedical research, medical education, and social interaction—it is essential to move towards experimental and evidence-based projects. We need robust empirical data to determine whether AI enhances or, conversely, undermines scientific work in the life sciences, and the extent to which it affects human physiology, mental health outcomes, psychomotor development, and overall quality of care.

Furthermore, the impact of social networks on individuals’ health perceptions and decision-making has emerged as a pivotal factor in clinical settings. Digital platforms frequently disseminate simplified, polarized, or misleading health information, thereby shaping users’ expectations and beliefs prior to any clinical encounter. This has the potential to compromise the doctor–patient relationship by introducing layers of bias and misinformation that can challenge effective communication and erode trust [20]. The potential of Artificial Intelligence in identifying such patterns of mediated influence is of particular significance, as it can provide the necessary tools for the detection and analysis of communication trends that distort medical knowledge and public opinion [21]. The aforementioned capabilities may assist clinicians in a deeper understanding of patients’ preconceptions, thereby facilitating more effective, transparent, and personalized care.

In light of these considerations, we propose the concept of a hybrid clinical methodology as a model for the integration of human clinical judgement and AI-driven systems. Rather than replace professional expertise, this approach aims to enhance it by fostering a dynamic interplay between experiential knowledge, ethical discernment, and machine-supported analysis. It encourages epistemological cooperation, whereby algorithms act as heuristic tools that are subject to continuous and critical validation and contextual interpretation. The model reflects a dynamic that operates from both the top down and the bottom up: foundational principles and normative criteria guide the use of technological tools, while empirical experience informs their refinement and adaptation to clinical realities.

While acknowledging the significance of regulations pertaining to informed consent in the context of Artificial Intelligence, it is imperative to investigate the actual impact of personalization algorithms on individual freedom. The crux of this debate pertains to the question of whether these technologies serve to enhance patient autonomy or whether they have the potential to erode it through the subtle fostering of influence or manipulation in decision-making processes.

In a similar vein, while the value of AI tools in medical education is acknowledged, the primary concern lies in the acquisition of empirical evidence to determine whether such tools genuinely support the development of critical and reflective thinking in future physicians or rather encourage a passive and standardized learning approach.

Recent real-world applications of AI in clinical practice highlight the opportunities and challenges posed by this technological shift. Several pilot studies have begun empirically assessing the effectiveness of AI tools in areas such as diagnostics, risk stratification, and medical education. For example, AI-based tools have demonstrated high accuracy in breast cancer screening when tested in realistic conditions, sometimes outperforming radiologists and reducing false positives and false negatives [22]. However, the deployment of the Epic Sepsis Model in US hospitals revealed the risks of relying on unvalidated proprietary algorithms, emphasizing the importance of transparent external evaluation prior to widespread implementation [23]. Similarly, a randomized controlled trial at the Angers School of Medicine in France found that students who used immersive virtual patient simulation (IVPS) alongside traditional coursework achieved significantly higher exam scores, reported greater engagement, and perceived greater realism than those who received traditional instruction alone [24]. These pilot applications highlight the potential of AI integration in healthcare and strengthen the case for systematic, evidence-based evaluation frameworks.

It is imperative to ensure universal access to AI-based technologies. However, we are even more committed to scientifically testing the hypothesis that these technologies, if driven by psycho-political objectives, may construct a non-prospective reality that fundamentally undermines individual freedom and erodes the democratic foundations of civil coexistence.

Reflections on this topic have reached a pivotal point in the revision of clinical deontology, as Artificial Intelligence (AI) challenges conventional responsibilities and reshapes the epistemological foundations of medical practice. The subsequent section undertakes an examination of the ethical and normative implications of this shift, with particular attention to hybrid methodologies and their experimental nature.

## 4. Artificial Intelligence and Medical Deontology: Towards an Ethical Protocol for Hybrid-Methodology-Based Therapy

The integration of Artificial Intelligence into the decision-making and operational processes of clinical practice raises a central and largely unexplored question: should a therapy based on hybrid methodology be subjected to a protocol similar to that required for experimental treatments?

This issue is not merely theoretical; it strikes at the core of the relationship between innovation, professional responsibility, and fundamental human rights. Therapies that employ generative, predictive, or adaptive AI—such as those used for tailoring diagnostic–therapeutic algorithms—alter the very nature of medical action. They introduce new forms of uncertainty and opacity, often unpredictable and not fully controllable by the clinician. In this sense, the boundary between “consolidated practice” and “technological experimentation” becomes thin and porous.

It is therefore necessary to address this challenge within a framework that provides effective safeguards for the patient, one that moves beyond the traditional physician-centered model and instead affirms the full centrality of the individual as a holder of fundamental rights, including health, freedom, dignity, transparency, and informed participation. This approach requires viewing any intervention involving AI tools as potentially experimental, to the extent that each individual’s biochemical, neurocognitive, and clinical profile may produce specific and non-fully modellable responses.

From this perspective, even the use of seemingly “assistive” tools—such as risk stratification software, automatically generated treatment plans, or decision support systems in complex cases—entails a calculated risk, comparable to dosage changes or off-label prescriptions. The margin of uncertainty, inherent to any clinical act, significantly expands when the cognitive foundation of the therapeutic process is partially delegated to an algorithmic system that is not fully transparent or interpretable, so-called black box AI [25]. If this is the case, the demarcation between ordinary medical acts and experimental treatments must be reconsidered—focusing not only on the therapeutic aim (which both share) but also on the degree of methodological validation, the traceability of decision-making processes, and the presence of a truly informed and conscious consent.

In particular, informed consent must undergo a profound conceptual and procedural redefinition. The major international bioethical declarations—from the World Medical Association’s Declaration of Helsinki [26] to the Council of Europe’s Oviedo Convention [27]—emphasize that consent cannot be a mere bureaucratic formality. It must result from a dialogical, multilayered process that accounts for the patient’s cultural, cognitive, and clinical specificities. In the context of AI, this means clearly explaining—through accessible, non-technical language—the algorithmic nature of the therapeutic support, its epistemic limitations, the system’s capacity for self-learning, and the potential for bias in recommendation generation.

The patient must understand not only what is being done but also how and by whom the proposed clinical options are generated. When facing algorithmic systems that are not entirely interpretable, the duty to inform cannot be limited to presenting expected benefits; it must also include the degree of uncertainty and the potential long-term unintended consequences. Furthermore, the responsibility relationship between physician and intelligent system must be explicitly stated, avoiding any implicit “displacement” of decisions onto technological entities devoid of moral agency.

The use of AI in medicine—particularly in high-complexity or high-vulnerability areas such as oncology, psychiatry, palliative care, or predictive medicine—must be conditioned on genuinely autonomous consent. This consent must be personalized, revisable over time, verifiable, documented, and revocable at any moment, in line with the European Union’s General Data Protection Regulation (GDPR) [28] and the emerging principle of “data dignity” at the international level. Following this logic, the adoption of hybrid methodology models—where collaboration between physician and AI is not neutral but epistemologically constitutive of the decision-making process—must be anchored in the *neminem laedere* principle and subordinated to the respect for personal freedom and dignity, as established in Article 32 of the Italian Constitution [29] and Article 3 of the Charter of Fundamental Rights of the European Union [30].

As in clinical experimentation, the use of advanced AI cannot rely on standardized consent procedures. It must consider the specific context, vulnerability, and cognitive capacity of the individual, avoiding both technocratic drift and paternalism. AI-based medicine calls for a participatory model in which patients become fully informed protagonists of the technological choices that affect them. Even in extreme conditions—such as terminal illnesses or untreatable diseases—the use of experimental technology is not ethically justified unless the patient’s informed will has been adequately evaluated and respected. The use of AI in such settings must be treated as any other innovative therapeutic act: legitimate only if directed toward a tangible, direct benefit for the patient, aligned with their values and treatment goals, and never imposed surreptitiously as a last resort in the absence of proper information.

Ultimately, the adoption of Artificial Intelligence in medicine must be governed by a fundamental deontological principle: no clinical act, however technologically advanced, can be justified if carried out without informed consent, under epistemic opacity, or in contradiction with the personalist principle that underpins all authentic care. In this context, the evolution of the Medical Deontology Code must necessarily incorporate the dimension of “Artificial Intelligence”, by establishing not only clear standards of accountability but also transparent and verifiable guidelines for the ethically acceptable clinical use of intelligent systems. The dual purpose is clear: to prevent AI from becoming a tool of de-responsibilization or automated decision-making and to ensure that the human person remains the inalienable center of every care process—even when mediated by Artificial Intelligences.

Although this proposal is grounded in the specific context of medical deontology, it is worth noting that various international guidelines and policy frameworks already provide valuable principles for the ethical use of AI in society. Notably, the European Union’s AI Act establishes a robust legal framework for risk-based regulation and the responsible integration of AI systems in sensitive areas such as healthcare [31]. While these references are not specific to medical ethics, they can inform and support the revision of the Code of Medical Deontology in an effort to include AI governance principles.

In order to provide a more solid foundation for this normative reflection, it is important to explore from a philosophical perspective how professional identity and clinical judgment are reshaped by technological mediation.

## 5. Philosophy and Professional Ethics: Outlines of Critical Medicine

In order to address the ethical and epistemological intricacies posed by AI and advanced technologies, medical practice must evolve into a form of critical medicine. This approach transcends abstract theorization, cultivating an ethos of perpetual reflection and personal accountability, firmly rooted in philosophical discernment.

People can lose their reflective-critical thinking for the sake of a flattening of appreciation, exposing them to the manipulation of social engineering and the deprivation of freedom as a consequence of the loss of self-awareness. This same phenomenon can be applied to health science and medical professions, where it is imperative to be cognizant of the direction or end of one’s actions, to discern when core values and ideals are being compromised, and to effectively manage external pressures to behave in a manner that is consistent with professional standards [32]. Accordingly, philosophical reflection plays a pivotal role in ensuring that medical practice remains profoundly human, conscious of its implications and committed to the pursuit of best practice, especially if these are properly conceived. This reflection should not be understood as an epistemological effort to reduce everything to an increasingly procedural methodology, nor as the application of philosophical visions to medicine. Rather, it constitutes a critical engagement that medicine itself undertakes with humanistic expertise and in collaboration with philosophers, addressing ontological and practical issues deemed necessary on a case-by-case basis. It is worth recalling the well-known saying of Hippocrates, “*Iatros philosophos isotheos*”, “the physician who becomes a philosopher is similar to a god”, which refers not to those who merely study some philosophy but rather to the physician who thinks and acts like a philosopher, that is, one who possesses a love of truth and a critical attitude. This statement may appear to be a vestige of a bygone era; however, the mounting challenges confronting medical professionals in their interactions with increasingly complex patients and scenarios, compounded by the integration of advanced technologies, underscores the necessity for medical practitioners to transcend their role as mere technicians. In this regard, the necessity to transition from a philosophy of medicine—which is expected to continue its progression—to a critical medicine approach is being asserted. This approach, when employed in conjunction with other medical competencies, facilitates the attainment of a multidimensional [33] and systemic understanding of the realities in which and for which physicians must engage. Critical medicine is not merely a philosophy of medicine but a reflective practice embedded within medical action, emphasizing awareness, accountability, and humanistic engagement. It entails constant questioning of its own premises and openness to new perspectives, while preserving a personalistic approach to care.

This general framework of critical medicine is rooted in two interconnected domains: the ethics of relationships and the impact of technological transformation. The following two key theses are formed on the basis of these domains.

The initial observation pertains to an inherent correlation between the quality and ethics of medical care and the quality of relationships across all levels of the healthcare ecosystem. These include, for example, the relationship between doctor and patient, doctor and colleagues, doctor and other health professionals, doctor and company, health system and family, medicine and society, medicine and policy makers, etc. This list could be extended to include other ramifications that are difficult to define, given the challenge of exhaustively mapping all the relationships in the health sector, which are manifold, interconnected, and constantly changing, according to a systemic and intersystemic logic. However, the quality, effectiveness, and efficiency of healthcare depends on the quality of each of these relationships. In terms of the ethics of the job well done, the first ethical duty of any professional is to do his or her job well. This means striving to fulfill the purpose of the work undertaken and acting in such a way as to achieve a synthesis between the immediate and ultimate ends. More specifically, since every action begins, develops, and ends in connection with other activities, a job well done will only be possible if each of the actions involved has been carried out correctly, i.e., properly completed. Returning to the topic under discussion, this requires that every relationship involved in the healthcare act be carefully managed and, more importantly, nurtured through a profound level of critical self-awareness of the personal dimension inherent in these relationships. Without this reflective capacity, which makes us aware of how our identity, emotions, desires, and beliefs can affect interactions with others, we risk building relationships that are inauthentic and disrespectful of others, for example, reducing the patient to an object, unconsciously acting according to power dynamics, or closing ourselves off in distorted communication. Examining the relational dimension of medical practice from a critical vantage point has the potential to steer medicine towards a personalized perspective that can enrich and integrate both the proceduralist and pragmatic approaches. This integration would be accompanied by a profound ontological and ethical understanding, thereby ensuring that treatment is not merely technical and effective but also person-centered and respectful of patient dignity. Consequently, medicine will no longer be confined to the application of standard procedures or the resolution of practical and concrete issues. Instead, it will evolve into a dynamic and adaptive process that acknowledges and responds to individual needs. This process will integrate scientific effectiveness with a realistic ethical–ontological perspective. In the context of Aristotle’s conception of friendship as founded on virtue [34], the cultivation of critical self-awareness concerning the personal dimension of relationships is instrumental not only to the objective fulfillment of one’s professional duties but also to the subjective aspect of that fulfillment. This underscores the potential of such self-awareness to serve as a catalyst for the personal, moral, and professional growth of healthcare workers.

The second point pertains to technology’s role in amplifying the necessity for critical, humanistic competencies and a renewed understanding of professional identity, rather than replacing the physician. This point necessitates an understanding of the nature of technology, something that has been employed by humans as rational beings capable of adapting to the environment—as Darwin explains [35], like other species—but also of adapting the environment to their own needs. In the context of Aristotelian thought, technology can thus be conceptualized as a ‘reinforcing alteration’ [36], signifying a learning process that facilitates the realization of a rational human being’s potential to a greater extent. Consequently, the increased utilization of technology in medicine would not result in an alteration to the status of the physician; rather, it would serve to strengthen the role of the doctor through ‘instrumental enhancement’. In order to facilitate the renewal of medical ethics in a manner that is responsive to these changes, it is imperative that the field adapts to technological progress in two distinct ways. Firstly, there is a necessity for amendments regarding the utilization of instrumental enhancement. Secondly, there is a requirement for the strengthening of the humanistic awareness of the status of the doctor. critical medicine refrains from any intimidation arising from the potential for destabilization or dehumanization through technological advancement, particularly within a de-structuralist paradigm. However, it is predicated on the reinforcement of its humanistic competencies to ensure the optimal governance of the desired and functional techniques, maintaining a careful balance between scientific innovation and the centrality of the person in the care process.

In light of the aforementioned backdrop, a critical approach to medicine is evolving as a means of responding to the necessity for reform and improvement, prompted by the inherent complexity and the effect of social, cultural, and scientific changes. It is, therefore, an ‘emergent reality’ insofar as it is growing and gaining relevance, thanks to the interaction of various factors, and reflects a shift in collective perceptions of medicine itself.

In considering complexity, the healthcare act must be regarded as an integrated process [37], whilst the medical act must be considered dynamically and systemically with respect to this same process. Previous attempts to define the medical act have been unsuccessful due to a lack of a global and interconnected perspective. It is therefore crucial for medical professionals to reflect on their professional behavior in this light in order to acquire self-awareness, which is an essential aspect of critical medicine. Consequently, there is an expectation that ethical codes will undergo revisions to incorporate the notion of the medical act as a fundamental and systemic component of the entire healthcare treatment process.

To summarize, these reflections advocate for a critical approach that reclaims the philosophical dimension of care as both practical and humanistic, preparing medicine to navigate the ethical complexities of an advanced technological context.

## 6. Discussion

Drawing upon the conceptual foundations previously outlined, this discussion explores the practical implementation of critical medicine, with a focus on the integration of knowledge systems, the evolving role of technology in decision-making, and the ethical centrality of human relationships.

The systemic perspective hereby articulated does not entail a shift in clinical methodology; rather, it supports the foundation for a multidimensional, integrated understanding of clinical practice—one that is cognizant of the inherent complexity of care. In this view, the traditional dualism between reductionism and anti-reductionism is transcended, giving way to a more realistic epistemological stance—one that acknowledges the authenticity, yet inherent partiality, of knowledge as it engages with the complexity of reality. However, such a form of knowledge must be understood as a collective endeavor. The principle of epistemological coherence among different fields of knowledge indicates the possibility—and indeed the necessity—of systematically relating the various forms of understanding developed by both theoretical and practical sciences around a shared object of inquiry. This approach involves an aware and cooperative reduction [38], in which disciplinary particularities are assimilated without being eradicated.

The integration of Artificial Intelligence within this process engenders a substantial transformation: the conventional notion of collective intelligence [39], based on connections among human minds (brain-to-brain), evolves into a hybrid collective intelligence, emerging from the interaction between human brains and AI systems (brain-to-brain-to-AI) [40]. This emerging configuration gives rise to a multitude of scenarios, both in terms of knowledge production and the ethical responsibilities that are inherent to knowledge creation.

From a theoretical perspective, this configuration may find an inspiring model in the dynamics of Generative Adversarial Networks (GANs) [41]. However, an alternative to the traditional competitive interaction between two Artificial Intelligences—characterized by a zero-sum logic—could be envisaged. This would be a cooperative paradigm involving two AIs and a human intelligence operating in synergy according to a Nash equilibrium logic [42]. In such a model, no participant has an incentive to unilaterally alter their strategy, thereby fostering convergence toward shared and sustainable solutions.

Within this framework, the Italian Code of Medical Deontology must evolve into a tool not only of regulation but of ethical reflection and relational maturity. In accordance with the ethics of the work well done, the Code could facilitate the integration of clinical methodology as a space for human and professional maturation. This methodology would be capable of addressing the challenges of medicine in an increasingly complex and technologically mediated cognitive ecosystem.

In addition to this perspective, the adoption of the notion of relational autonomy offers a robust ethical foundation for navigating medical decisions within socially and technologically complex contexts. Rather than conceptualizing autonomy as merely individual self-determination, relational autonomy underscores the social and interpersonal dimensions of decision-making. It acknowledges the inherent interconnectedness of individuals within networks of relationships and posits that autonomy is realized through the medium of dialogue, mutual recognition, and contextual support [43]. This concept is in alignment with the ethical complexities of modern clinical practice, where decisions are rarely made in isolation and are increasingly mediated by technological and organizational factors. This approach facilitates a reinterpretation of fundamental concepts such as informed consent, professional secrecy, and the principle of proportionality, thereby enhancing the dialogic dynamic between patient and doctor. Indeed, it is within the context of relationships that individuals are able to make decisions, to recognize themselves as persons, and to act accordingly.

This is the point at which consideration of the relationship between subject and technology becomes pertinent, facilitating the transcendence of the reductive dichotomy between subject and object. The utilization of technology is never impartial. It involves the subject as such and contributes, over time, to shaping personal and collective consciousness. This critical and relational perspective provides a more robust foundation for the code of medical ethics, capable of encompassing the complexity of clinical decisions, the ethical dimensions of medical practice, and the expanding influence of technology on healthcare delivery and its associated cultural practices.

These theses come together towards a model of critical medicine that brings technology, ethics, and human relationships within a coherent framework, capable of addressing the evolving challenges of contemporary care.

## 7. Conclusions

Although this reflection has focused primarily on the Italian Code of Medical Deontology, a comparative analysis with other national or international ethical frameworks could inform future developments. The challenges posed by Artificial Intelligence to clinical practice—particularly regarding responsibility, transparency, and informed consent—are not confined to a single jurisdiction. This underscores the importance of developing shared international approaches to avoid disparities in patient care and to support consistent ethical standards across healthcare systems.

In this context, AI-informed therapeutic interventions, due to their novel and potentially uncertain nature, may necessitate protocols akin to those governing experimental treatments. Such protocols would likely necessitate oversight by institutional review boards or ethics committees to ensure patient safety, transparency, and informed consent. From a legal standpoint, compliance with existing regulations such as the GDPR is paramount, particularly regarding the processing of sensitive health data (including data minimization, purpose limitation, and explicit consent). Moreover, the emergence of frameworks such as the EU’s proposed AI Act may result in the introduction of additional requirements pertaining to risk assessment and accountability in the context of AI-driven healthcare. The integration of AI-informed interventions within the established legal and ethical frameworks is imperative to facilitate responsible innovation while ensuring the safeguarding of patient welfare and autonomy.

## Data Availability

Data is contained within the article.
